# Mortalidade Hospitalar em Pacientes com Choque Cardiogênico após Infarto do Miocárdio: Há Benefício no Uso de Balão Intra-Aórtico?

**DOI:** 10.36660/abc.20250080

**Published:** 2025-02-27

**Authors:** Fernando Arturo Effio Solis, Adriana Brentegani, Marcelo Luiz Campos Vieira

**Affiliations:** 1 Hospital Israelita Albert Einstein São Paulo SP Brasil Hospital Israelita Albert Einstein, São Paulo, SP – Brasil; 2 Hospital Sírio-Libanês São Paulo SP Brasil Hospital Sírio-Libanês, São Paulo, SP – Brasil; 3 Instituto do Coração do Hospital das Clínicas Faculdade de Medicina Universidade de São Paulo São Paulo SP Brasil Instituto do Coração do Hospital das Clínicas da Faculdade de Medicina da Universidade de São Paulo, São Paulo, SP – Brasil

**Keywords:** Choque Cardiogênico, Balão Intra-aórtico, Infarto do Miocárdio com Supradesnível do Segmento ST, Mortalidade Hospitalar

O choque cardiogênico (CC) representa uma síndrome clínica grave caracterizada por hipoperfusão sistêmica e débito cardíaco insuficiente devido à disfunção cardíaca primária. Sua mortalidade frequentemente ultrapassa 40%^1 , 2^ dos casos, sendo uma condição cardiológica de alta complexidade. A etiologia é multifatorial, sendo o infarto agudo do miocárdio (IAM) a causa predominante, responsável por cerca de 30% dos casos.^2^ Outras condições relevantes incluem descompensação aguda da insuficiência cardíaca crônica e casos de disfunção miocárdica aguda sem história prévia de insuficiência cardíaca, como observado na miocardite.^3^

O manejo do CC pós-IAM requer uma abordagem abrangente, que combine revascularização precoce e suporte hemodinâmico.^4 - 7^ Nesse contexto, dispositivos como o balão intra-aórtico (BIA) têm seu papel devido à sua capacidade de aumentar a pressão diastólica na aorta e reduzir a pós-carga ventricular esquerda.^8^ Esses efeitos fisiológicos resultam em melhora da perfusão coronária e redução do consumo de oxigênio pelo miocárdio.

Entretanto, estudos recentes não demonstraram benefícios significativos do BIA em termos de redução da mortalidade, levando a uma revisão de seu papel em diretrizes internacionais.^9^ Essas evidências contribuíram para a redução de seu uso em muitos centros, reforçando a necessidade de análise cuidadosa na seleção de pacientes que podem se beneficiar.

Assim, pesquisadores nacionais, em um estudo observacional transversal, conduziram uma análise retrospectiva de 98 pacientes de um único centro médico, analisando pacientes acometidos por IAM com supradesnivelamento do segmento ST (IAMCSST) e CC tratados com BIA,^10^ com o objetivo de avaliar possíveis preditores da eficácia do BIA na redução da mortalidade hospitalar.

Noventa e oito pacientes foram selecionados de 2005 a 2022. A amostra foi composta principalmente por homens (73,5%) com média de idade de 66,5 ± 12,3 anos. A hipertensão arterial sistêmica foi a comorbidade mais prevalente (73,7%), seguida de tabagismo (37,8%) e dislipidemia (46,9%).

A maioria dos pacientes encontrava-se em estágio avançado de gravidade (Killip IV, 39,2%) no momento da admissão, e quase todos apresentavam disfunção ventricular (95,9%), sendo a artéria descendente anterior identificada como a mais frequentemente acometida (80%). O uso do BIA no mesmo dia do IAM foi prevalente (74,5%), com a maioria dos dispositivos sendo utilizados por três ou mais dias (46,9%). O percentual total de óbitos atingiu 43,9% e 55,7% de altas hospitalares. Não foram observadas associações significativas entre a maioria dos fatores clínicos e demográficos avaliados (sexo, tempo porta-balão, IAM prévio, comorbidades, entre outros) e o risco de óbito (p ≥ 0,05). Pacientes que utilizaram o dispositivo por dois ou mais dias apresentaram menor risco de óbito em comparação àqueles que o utilizaram por apenas 0-1 dia. Fatores como idade e dislipidemia mostraram-se preditores independentes no modelo multivariado para o desfecho primário de óbito hospitalar. Cada ano adicional de idade aumentou a chance de morte em 7% (OR 1,07; p = 0,005), enquanto a presença de dislipidemia foi associada a um efeito protetor (OR 0,21; p = 0,005). Observou-se também que os pacientes que receberam o BIA um dia após o IAM apresentaram menor risco de morte (OR 0,05; p = 0,002) em comparação aos que receberam o dispositivo no dia 0.

A descrição metodológica da investigação é adequada e detalhada, com aplicação de análise estatística apropriada, permitindo identificar potenciais associações relevantes, como a influência do tempo de uso e do momento de implementação do BIA na mortalidade hospitalar. A seção de limitações do manuscrito destaca as limitações do estudo (estudo realizado a partir de prontuários médicos, alterações temporais nos critérios de CC) e a consideração de um pequeno número de pacientes envolvidos na investigação, o que reduz o poder estatístico e a reprodutibilidade dos achados, com consequente redução da força das conclusões. Além disso, por se tratar de um estudo retrospectivo e unicêntrico, generalizar os resultados para outras populações e diferentes contextos clínicos torna-se desafiador.

Outro ponto crítico é a ausência de subanálises estratificadas por subgrupos, o que poderia fornecer insights adicionais sobre quais perfis de pacientes se beneficiam mais do BIA. A inclusão de pacientes com mais de 17 anos pode ter introduzido viés temporal, pois mudanças nas diretrizes clínicas, na disponibilidade de tecnologias e na abordagem terapêutica do CC podem ter impactado os resultados ao longo do período estudado.

Além disso, o estudo não aborda possíveis complicações associadas ao uso do BIA, como infecção, trombose ou eventos hemorrágicos, que são fatores críticos na evolução do paciente e que podem estar implicados no aumento da mortalidade hospitalar.

Em resumo, embora o BIA possa proporcionar benefícios hemodinâmicos em determinados cenários, evidências atuais requerem estudos com maior número de pacientes e de natureza multicêntrica para embasar seu uso rotineiro na redução da mortalidade em pacientes com CC pós-IAM. No entanto, é um dispositivo relativamente simples, de menor custo e mais amplamente disponível do que outras opções, como a ECMO. Portanto, sua indicação deve ser baseada em uma avaliação criteriosa do estado clínico do paciente e das alternativas de suporte circulatório mecânico disponíveis.

Portanto, estudos futuros, preferencialmente prospectivos, multicêntricos e com maior número de participantes, são necessários para validar esses achados e refinar as indicações do BIA na prática clínica. Parabenizamos também os autores brasileiros pela iniciativa da investigação e pela publicação de seus resultados.
