# Supplementary material for: Mortalidade Hospitalar em Pacientes com Choque Cardiogênico após Infarto do Miocárdio: Há Benefício no Uso de Balão Intra-Aórtico?
Source: Arq Bras Cardiol. 2025 Feb 27;122(2):e20250080. [Article in Portuguese] doi: 10.36660/abc.20250080 (PMC12080612; doi:10.36660/abc.20250080)
Supplement: Translation

# Translation: In-Hospital Mortality in Patients Presenting Cardiogenic Shock After Myocardial Infarction: Does it Benefit Using an Intra-Aortic Balloon Pump?

**DOI:** 10.36660/abc.20250080i

**Authors:** Fernando Arturo Effio Solis, Adriana Brentegani, Marcelo Luiz Campos Vieira

**Affiliations:** 1 Brasil Hospital Israelita Albert Einstein, São Paulo, SP – Brasil; 2 Brasil Hospital Sírio-Libanês, São Paulo, SP – Brasil; 3 Brasil Instituto do Coração do Hospital das Clínicas da Faculdade de Medicina da Universidade de São Paulo, São Paulo, SP – Brasil

**Keywords:** Cardiogenic Shock, Intra-aortic Balloon, ST-Segment Elevation Myocardial Infarction, In-Hospital Mortality

Cardiogenic shock (CS) represents a severe clinical syndrome characterized by systemic hypoperfusion and insufficient cardiac output due to primary cardiac dysfunction. Its mortality often exceeds 40%^1 , 2^of cases, being a highly complex cardiological condition. The etiology is multifactorial, with acute myocardial infarction (AMI) being the predominant cause, responsible for around 30% of cases.^2^Other relevant conditions include acute decompensation of chronic heart failure and cases of acute myocardial dysfunction without a previous history of heart failure, as observed in myocarditis.^3^

The management of post-AMI CS requires a comprehensive approach, which combines early revascularization and hemodynamic support.^4 - 7^In this context, devices such as the intra-aortic balloon pump (IABP) have their role due to their ability to increase diastolic pressure in the aorta and reduce left ventricular afterload.^8^These physiological effects result in improved coronary perfusion and reduced myocardial oxygen consumption.

However, recent studies have not demonstrated significant benefits of IABP in terms of reducing mortality, leading to a review of its role in international guidelines.^9^This evidence has contributed to the reduction in its use in many centers, reinforcing the need for careful analysis in the selection of patients who can benefit.

Thus, national researchers in an observational, cross-sectional study conducted a retrospective analysis on 98 patients from a single medical center, analyzing patients affected by ST-segment elevation AMI (STEMI) and CS treated with IABP,^10^with the aim of evaluating possible predictors of IABP effectiveness in reducing in-hospital mortality.

Ninety-eight patients were selected from 2005 to 2022. The sample was mainly composed of men (73.5%) with a mean age of 66.5 ± 12.3 years. Systemic arterial hypertension was the most prevalent comorbidity (73.7%), followed by smoking (37.8%) and dyslipidemia (46.9%).

Most patients were in an advanced stage of severity (Killip IV, 39.2%) at the time of admission, and almost all had ventricular dysfunction (95.9%), with the anterior descending artery identified as the most frequently affected (80 %). The use of IABP on the same day as the AMI was prevalent (74.5%), with most devices being used for three or more days (46.9%). The total percentage of deaths reached 43.9% and 55.7% of hospital discharges. No significant associations were observed between most of the clinical and demographic factors assessed (gender, door-to-balloon time, previous AMI, comorbidities, among others) and the risk of death (p ≥ 0.05). Patients who used the device for two or more days had a lower risk of death compared to those who used it for only 0-1 day. Factors such as age and dyslipidemia proved to be independent predictors in the multivariate model for the primary outcome of in-hospital death. Each additional year of age increased the chance of death by 7% (OR 1.07; p = 0.005), while the presence of dyslipidemia was associated with a protective effect (OR 0.21; p = 0.005). It was also observed that patients who received the IABP one day after the AMI had a lower risk of death (OR 0.05; p = 0.002) compared to those who received the device on day 0.

The methodological description of the investigation is adequate and detailed, with the application of appropriate statistical analysis, allowing the identification of potential relevant associations, such as the influence of time of use and the moment of IABP implementation on in-hospital mortality. The limitations section of the manuscript highlights the study’s limitations (study carried out from medical records, temporal changes in CS criteria) and the consideration of a small number of patients involved in the investigation, which reduces the power of statistical and reproducibility of the findings, with a consequent reduction in the strength of the conclusions. Furthermore, as this is a retrospective and single-center study, generalizing the results to other populations and different clinical contexts becomes challenging.

Another critical point is the absence of subanalyses stratified by subgroups, which could provide additional insights into which patient profiles benefit most from IABP. The inclusion of patients over 17 years may have introduced temporal bias, as changes in clinical guidelines, the availability of technologies, and the therapeutic approach to CS may have impacted outcomes throughout the period studied.

Furthermore, the study does not address possible complications associated with the use of IABP, such as infection, thrombosis, or bleeding events, which are critical factors in the patient’s evolution and which may be implicated in increased in-hospital mortality.

In summary, although the IABP can provide hemodynamic benefits in certain scenarios, current evidence requires studies with a larger number of patients and of a multicenter nature to support its routine use to reduce mortality in patients with post-AMI CS. However, it is a relatively simple device, lower in cost, and more widely available than other options, such as ECMO. Therefore, its indication must be based on a careful assessment of the patient’s clinical status and the available mechanical circulatory support alternatives.

Therefore, future studies, preferably prospective, multicenter, and with a larger number of participants, are necessary to validate these findings and refine the indications for IABP in clinical practice. We also congratulate the Brazilian authors for the initiative of the investigation and the publication of their results.
